# Isolation and molecular characterization of subgroup J avian leukosis virus in native chicken breeds of China during 2022–2025

**DOI:** 10.3389/fmicb.2025.1684812

**Published:** 2025-10-06

**Authors:** Qi Liu, Sifan Ji, Minghui Li, Lei He, Ke Ding, Zuhua Yu, Jian Chen

**Affiliations:** ^1^College of Animal Science and Technology/Laboratory of Functional Microbiology and Animal Health, Henan University of Science and Technology, Luoyang, China; ^2^Luoyang Key Laboratory of Functional Microbiology and Animal Health, Luoyang, China; ^3^The Key Laboratory of Animal Disease and Public Health, Henan University of Science and Technology, Luoyang, China; ^4^College of Animal Science and Veterinary Medicine, Henan Institute of Science and Technology, Xinxiang, China

**Keywords:** ALV-J, molecular characteristic, 3′ untranslated region, Gp85, phylogenetic analysis

## Abstract

Avian leukosis virus subgroup J (ALV-J) primarily infects poultry, especially chickens, where it induces immunosuppression and tumorigenesis. ALV-J has caused substantial economic losses worldwide and is prevalent among indigenous chicken breeds in China. In this study, we analyzed the genomic characteristics of ALV-J strains isolated from diseased liver tissue or anticoagulant blood samples collected from Lushi chickens, Central Plains cockfighting, and Hetian chickens between 2022 and 2025. The results showed that the nine isolates clustered within Clades 1.2 and 1.3, indicating that ALV-J is concurrently prevalent in multiple native chicken lineages. Compared with the ALV-J prototype strain HPRS-103, multiple specific functionally significant point mutations or deletion mutations occurred in the Gp85 protein and the 3′ untranslated region (3′ UTR) of all the isolates. These included the N123I mutation in the Gp85 protein, which stabilizes the Gp85 structure and expands the interaction interface, the D191N mutation suggesting the formation of a new N-glycosylation site, and the deletion mutations within the receptor-binding domain (RBD) that affect the efficiency of the binding between the virus and host cell receptors, as well as the reduced transmembrane (rTM) deletion mutation in the 3′ UTR that influences the viral replication ability, suggesting that the isolates analyzed may exhibit enhanced replication ability and pathogenicity. In addition, there are certain differences in the number of α-helices in the Gp85 proteins of these ALV-J strains, and these differences may have an impact on the interaction between the virus and host. The results of our study are conducive to enriching the epidemiological data of ALV-J and revealing the genetic evolution direction of ALV-J strains, which will provide a scientific basis for the prevention and control of avian leukosis.

## 1 Introduction

Avian leukosis virus (ALV) is an oncogenic and immunosuppressive retrovirus that causes reduced fertility, growth retardation, and increased susceptibility to secondary infections in poultry (Payne and Nair, 2012; Feng et al., 2017). Since its discovery, ALV has caused substantial economic losses globally. Although ALVs have been nearly eradicated from commercial chicken breeds in Western countries, they continue to circulate in many indigenous Chinese chicken breeds and pose a latent threat to other regions as potentially pathogenic agents (Malhotra et al., 2015; Zheng et al., 2022; Deng et al., 2022). ALVs are classified into 11 subgroups (A–K) according to host range, Gp85 (SU) envelope glycoprotein sequence, and overall genomic characteristics. Endogenous ALV is mainly represented by ALV-E, which is nonpathogenic. Among the exogenous ALVs, ALV-J, ALV-K, and ALV-A are the main prevalent subgroups (Wang et al., 2025; Zhang et al., 2024; Zhang et al., 2025).

ALV is an *Alpha-Retrovirus*, with a genome of approximately 7.2 ~ 7.8 Kb in size and a structure configured as 5′-LTR-gag-pol-env-LTR-3′ (Fandiño et al., 2023). The *gag* gene encodes internal proteins, including matrix protein (MA), p10, nucleocapsid protein (NC), protease (PRO), as well as an antigenic capsid protein p27 (CA) that is common to all subgroups of ALV (Freick et al., 2022). The *pol* gene encodes the proteins required for the replication of ALV (Su et al., 2018). The *env* gene encodes the envelope protein, which can be divided into two parts: the surface protein Gp85 and the transmembrane protein Gp37 (TM) (Fandiño et al., 2023). The Gp37 anchors the Gp85 to the viral envelope, enabling the proteins to be correctly positioned and facilitating their roles in viral infection and membrane fusion (Mothes et al., 2000). The host range regions 1 and 2 (hr1 and hr2) within Gp85 primarily determine the receptor specificity of ALVs, while the variable regions 1, 2, and 3 (vr1, vr2, and vr3) represent secondary sites of variation among ALV strains, notably, vr3 contributes to the specificity of receptor interactions (Chen et al., 2022; Chen et al., 2020). The long terminal repeat (LTR) plays a critical role in viral oncogenesis by activating host proto-oncogenes such as c-myc (Fung et al., 1983). It consists of three functionally distinct regions: U3, which contains enhancer elements and a promoter, R, which marks the transcription start site, and U5, which possesses post-translational regulatory elements and contribute to polyadenylation (Böhnlein et al., 1989; Ruddell, 1995). In contrast to the conserved U5 region, the U3 region exhibits considerable variability.

Avian leukosis virus subgroup J (ALV-J) is the causative agent of myeloid leukosis and was first identified in broiler chickens in England in 1988 (Payne et al., 1991). Subsequently, ALV-J was first isolated from broiler flocks in China in 1999, after which it rapidly spread to layer chickens and various indigenous breeds (Du and Ai, 1999; Xu et al., 2004; Sun and Cui, 2007). ALV-J infection leads to persistent high-level viremia in broiler chickens throughout their lifespan (Fadly and Smith, 1999). Due to the extremely high rates of vertical and horizontal transmission, ALV-J exhibits substantial genetic and antigenic variations among isolates, which complicates efforts at eradication (Meng et al., 2022; Li et al., 2017; Liao et al., 2022; Liu et al., 2021). The C terminus of Gp37 plays a crucial role in the pathogenesis of ALV-J (Li et al., 2020). The Gp85 of ALV-J exhibits high variability and undergoes rapid adaptive evolution under the selective pressure of host resistance induced by biotechnological approaches, which poses a significant challenge to develop ALV-J resistance through gene editing (Meng et al., 2016; Matoušková et al., 2024). Based on the genetic sequence variations in *gp85*, ALV-J can be further classified into Clades 1.1, 1.2, 1.3, 2, and 3 (Deng et al., 2022).

The 3′ untranslated region (3′ UTR) of ALV-J is one of the principal regions for viral mutation and evolution, and it also serves as a crucial component in regulating the viral life cycle and pathogenicity. The 3′ UTR of the ALV-J prototype strain HPRS103 is composed of four regions: r-TM, DR-1, E element and U3. Compared with HPRS103, ALV-J strains isolated after 1988 showed varying degrees of deletions in the 3′ UTR. However, the functions of the ALV-J 3′ UTR are not yet fully understood. Current studies have shown that r-TM is associated with the pathogenicity of ALV-J in laying hens, and its deletion significantly enhances the replication ability of ALV-J strains (Wang et al., 2012; Xu et al., 2023). DR-1 is essential for the export of viral mRNA from the nucleus (Ogert et al., 1996). The E element is closely associated with the virus’s oncogenicity and replication in poultry (Chesters et al., 2006).

In this study, nine strains of ALV-J were isolated from Lushi chicken and Central Plains Cockfighting in Henan province and Hetian chicken in Fujian province, China. The strains were designated HN22LS01, HN24HT01, HN24HT02, HN24HT03, HN24HT04, HN24HT05, HN25DJ01, HN25DJ02, and HN25DJ03, and sequence homology analysis, phylogenetic tree analysis, characteristic mutation analysis and protei structure prediction were carried out on the gp85 gene, and characteristic mutation analysis was performed on the 3′UTR and the LTRU3 region.

## 2 Methods and materials

### 2.1 Clinical samples

During the period from 2022 to 2025, samples were collected from three farms in China. Specifically, the deceased Lushi chicken from a breeding company with a scale of 20,000 chickens in Lushi County, Henan Province, was found to have an enlarged liver with grayish-white necrotic spots after post-morexamination; anticoagulant blood samples were collected from Hetian chickens at a breeding company with a stock of 30,000 breeding chickens in Fujian Province. Virus isolation and detection using DF-1 cells showed that some samples were positive for ELISA p27; 2 batches of liver samples of deceased fighting chickens were sourced from an individual breeder with a breeding scale of about 100 chickens in Shangqiu, Henan Province. And post-morexamination revealed that the chickens’ livers were abnormally enlarged and filled the abdominal cavity.

### 2.2 Virus isolation and proviral DNA extraction

DF-1 cells were cultured in DMEM (Servicebio, Wuhan, China) supplemented with 10% FBS (Clark Bioscience, Shanghai, China), and 2% penicillin–streptomycin at 37 °C under 5% CO₂. Liver tissue (0.1 g) was homogenized and centrifuged at 3,000 rpm for 10 min at 4 °C. The supernatant was filtered through a 0.22 μm filter. Serum samples were processed in the same manner. Filtered supernatants and sera were inoculated onto DF-1 monolayers. After 2 h incubation at 37 °C, the medium was replaced with DMEM containing 1% FBS. Infected cells were cultured for 7 days. After infection, cells underwent one freeze–thaw cycle (−80 °C to 37 °C), were centrifuged at 2,000 rpm for 10 min at 4 °C, and genomic DNA was extracted from the pellet using a commercial kit according to the manufacturer’s instructions.

### 2.3 PCR and sequencing

PCR amplification was performed using ALV-A-specific primers, yielding a 694 bp product for ALV-A detection. PCR amplification was performed using ALV-B-specific primers, yielding a 1,100 bp product for ALV-B detection. PCR amplification was performed using ALV-K-specific primers, yielding a 1,214 bp product for ALV-K detection. PCR amplification was performed using ALV-J-specific primers, yielding a 545 bp product for ALV-J detection. Four primer pairs covering the entire ALV-J genome were designed for whole-genome amplification (Table 1). The 648-bp PCR product was amplified using 2 × Taq Master Mix (Vazyme, Nanjing, China), while high-fidelity DNA polymerases were used for the remaining three genomic fragments. The thermal cycling conditions for the 648-bp fragment were: initial denaturation at 95 °C for 5 min; followed by 35 cycles of denaturation at 95 °C for 30 s, annealing at 55 °C for 30 s, and extension at 72 °C for 40 s; with a final extension at 72 °C for 8 min. The cycling parameters for the other three genomic regions were: initial denaturation at 95 °C for 3 min; followed by 30 cycles of denaturation at 95 °C for 15 s, annealing at 60 °C for 15 s, and extension at 72 °C for 2 min. The amplification products were subjected to 1% agarose gel electrophoresis and visualized using a gel imaging system. The amplification products were recovered using the StarPrep DNA Gel Extraction Kit (GenStar, Beijing, China), and then ligated into the pMD-19 T vector. Individual clones were selected and subjected to gene sequencing by Beijing Tsingke Biotechnology Co., Ltd. The env genes of HN25DJ02, HN24HT02, HN24HT03, HN24HT04, and HN24HT05 were successfully sequenced. Subsequently, the whole-genome assembly of HN25DJ01, HN25DJ03, HN22LS01, and HN24HT01 was completed using the DNASTAR SeqMan software.

**Table 1:** Primers for detection of ALV-J and primers for PCR amplification of ALV-J complete genome.

Primer	Sequences(5′−3′)	PCR product size
J545-F	GGATGAGGTGACTAAGAAAG	545 bp
J545-R	CGAACCAAAGGTAACACACG	
S1-F	TGTAGTGTTATGCAATACTCTTATG	2,457 bp
S1-R	CCTCCTCTGAAATAATAGTGATG	
S2-F	CATCACTATTATTTCAGAGGAGG	2,806 bp
S2-R	CCTCATCTTTCTTAGTCACC	
S3-F	GATGAGGCGAGCCCTCTCTTTG	2,200 bp
S3-R	TGTGGTGGGAGGTAAAATGGCGT	
S4-F	GAATTAGGGAGCAGCTGTAG	648 bp
S4-R	TGAAGCCTTCTGCTTCATTCAGG	
A-F	GGATGAGGTGACTAAGAAAG	694 bp
A-R	AGAGAAAGAGGGGYGTCTAAGGAGA	
K-F	TCCAGGCCGCAACTCAC	1,214 bp
K-R	CATACCACCACCCACGTACT	
B-F	CGAGAGTGGCTCGCGAGATGG	1,100 bp
B-R	AGCCGGACTATCGTATGGGGTAA	

### 2.4 Sequence alignment and phylogenetic analysis

Sequence alignment and homology analysis were conducted using the Clutal W Method in DNASTAR MegAlign. Phylogenetic analysis was performed with the neighbor-joining method in Molecular Evolutionary Genetics Analysis version 12 (MEGA 12), with 1,000 bootstrap replicates. All the reference sequences were obtained from GenBank and shown in Supplementary Table S1.

### 2.5 Secondary structure prediction and analysis of ALV-J Gp85 protein

The secondary structure of ALV-J Gp85 protein was predicted by AlphaFold 3,^1^ and the software PyMOL was used for visual analysis of the protein structure (Abramson et al., 2024).

### 2.6 Nucleotide sequence accession numbers

The sequences obtained in this study have been submitted to GenBank, and the accession numbers are provided in Table 2.

**Table 2:** Detailed information on the ALV-J isolated in this study.

Strains	Origin	Year	Host	Accession no.
HN22LS01	Henan	2022	Lushi chicken	PX099218
HN24HT01	Fujian	2023	Hetian chicken	PX099219
HN24HT02	Fujian	2023	Hetian chicken	PX108887
HN24HT03	Fujian	2023	Hetian chicken	PX108888
HN24HT04	Fujian	2023	Hetian chicken	PX108889
HN24HT05	Fujian	2023	Hetian chicken	PX108890
HN25DJ01	Henan	2025	Cockfighting	PX099220
HN25DJ02	Henan	2025	Cockfighting	PX108891
HN25DJ03	Henan	2025	Cockfighting	PX099221

## 3 Results

### 3.1 Isolation and identification of ALV-J strains

**Figure 1:**
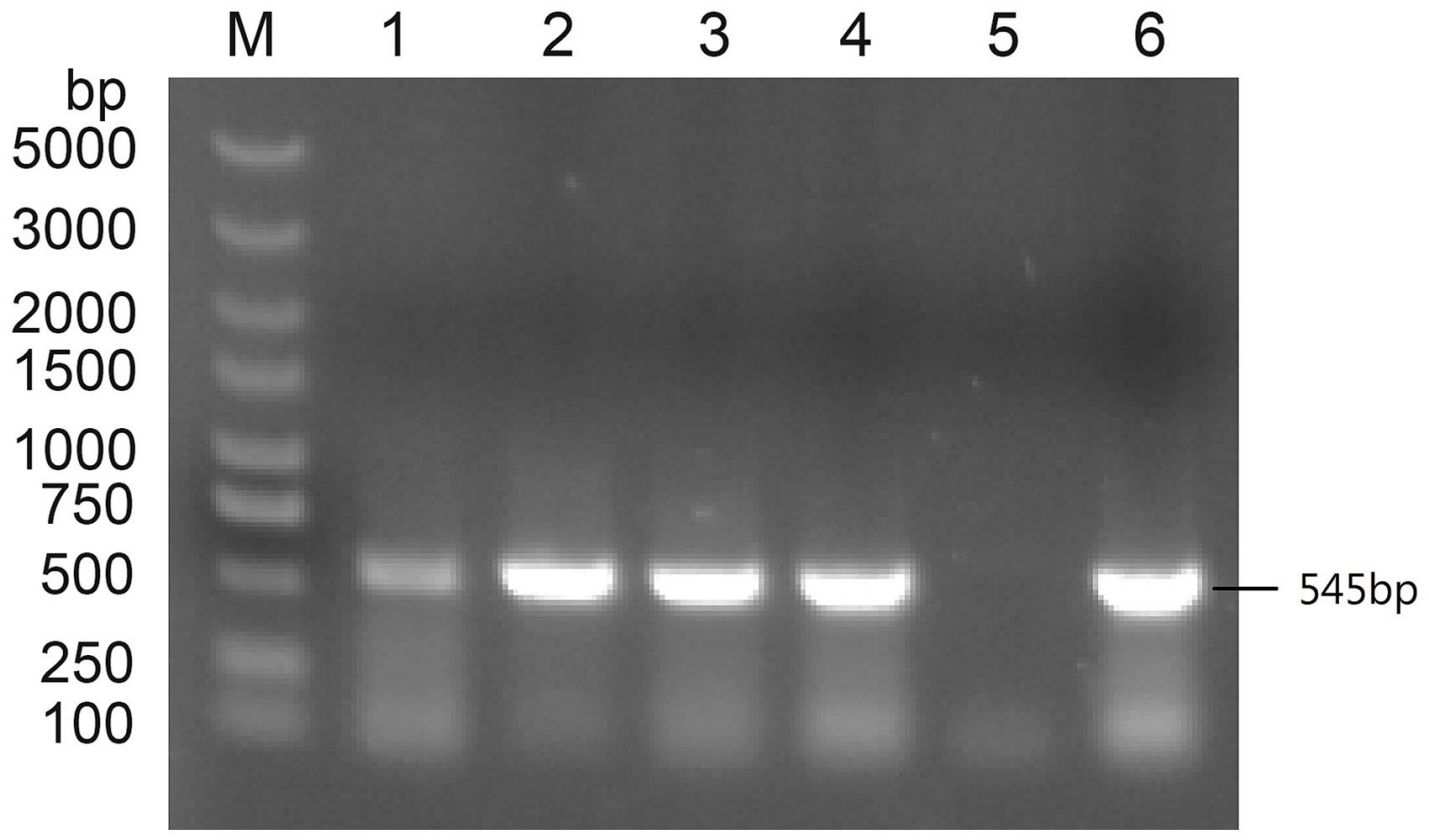
The PCR result of primers specific targeting ALV-J. M: Maker, 1: DNA of liver samples from Lushi chickens, 2: Isolate serum from anticoagulated blood to infect DF-1 cells, 3: DNA of liver samples from the first batch of cockfighting, 4: DNA of liver samples from the second batch of cockfighting, 5: Negative control, 6: Positive control.

**Figure 2:**
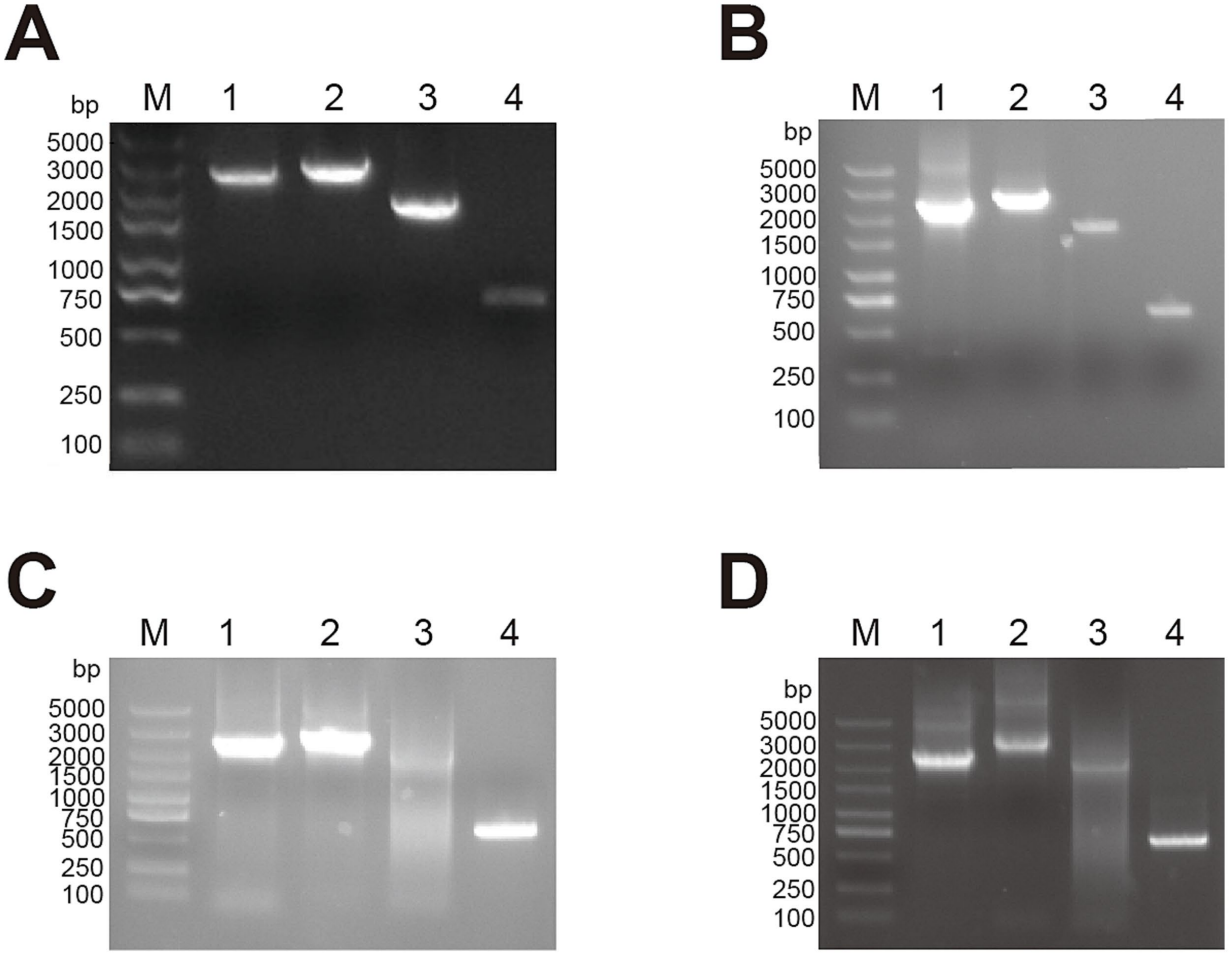
The PCR result of full-length primers specific for ALV-J. M: Maker, 1: 2,457 bp amplification product, 2: 2,806 bp amplification product, 3: 2,200 bp amplification product, 4: 648 bp amplification product. **(A)** HN22LS01 **(B)** HN24HT01 **(C)** HN25DJ01 **(D)** HN25DJ03.

Subgroup-specific detection was performed on the clinical samples submitted by various farms to detect AIV. The ALV-J specific bands of approximately 545 bp were successfully amplified (Figure 1). When amplified using the primers specific for ALV-A and ALV-B, no bands were amplified from all the samples. When amplified using the primers specific for ALV-K, no bands were amplified from the samples of cockfighting. However, the samples of Lushi chickens and Hetian chickens were amplified to yield specific bands with a length of 1,214 bp (Supplementary Figure S1). Subsequently, the tissue homogenates or sera that were detected as ALV-J positive were inoculated into DF-1 cells for virus isolation and identification. ELISA results showed that the DF-1 cells inoculated with PCR-positive samples were all ALV-positive. Four pairs of primers were used to perform PCR amplification on four ALV-J isolates. The results showed that all four ALV-J isolates successfully amplified the four target fragments (Figure 2). The amplified fragments were ligated into the pMD-19T vector, sequenced, and assembled to obtain the complete genomic sequences of the four ALV-J isolates, which were named HN22LS01, HN24HT01, HN25DJ01, and HN25DJ03, with genome lengths of 7,609, 7,582, 7,609, and 7,645 nt, respectively. Additionally, five ALV-J isolates were obtained. These five isolates were named HN24HT02, HN24HT03, HN24HT04, HN24HT05, and HN25DJ02 respectively, and their *env* genes were also obtained at the same time, with env sequence lengths of 1,655, 1,649, 1,652, 1,653, and 1,683 nt, respectively. All obtained sequences were consistent with the characteristic features of ALV.

### 3.2 Phylogenetic and homology analysis the ALV-J isolates

The full-lengths genomes of the four ALV-J isolates ranged from 7,582 to 7,645 bp. Compared with the whole genome sequences of the reference strains, the sequence homology ranged from 93.7 ~ 95.9%. Compared with the ALV-J strains that have been published in GenBank, the *pol* gene sequences of the four ALV-J strains with full-length genomes were highly conserved, with a homology ranging from 96.2 ~ 98.9%. The homology of the *gag* gene was from 93.6 ~ 96.4%, and that of the LTR was from 84.8 ~ 95.7%. Compared with the ALV-J strains published in GenBank, the *env* gene homology of the 9 ALV-J strains was from 91.1% ~ 96.5%, the *gp85* gene homology was from 85.5 ~ 96.8%, and the *gp37* gene homology was from 91.9% ~ 97.9%.

**Figure 3:**
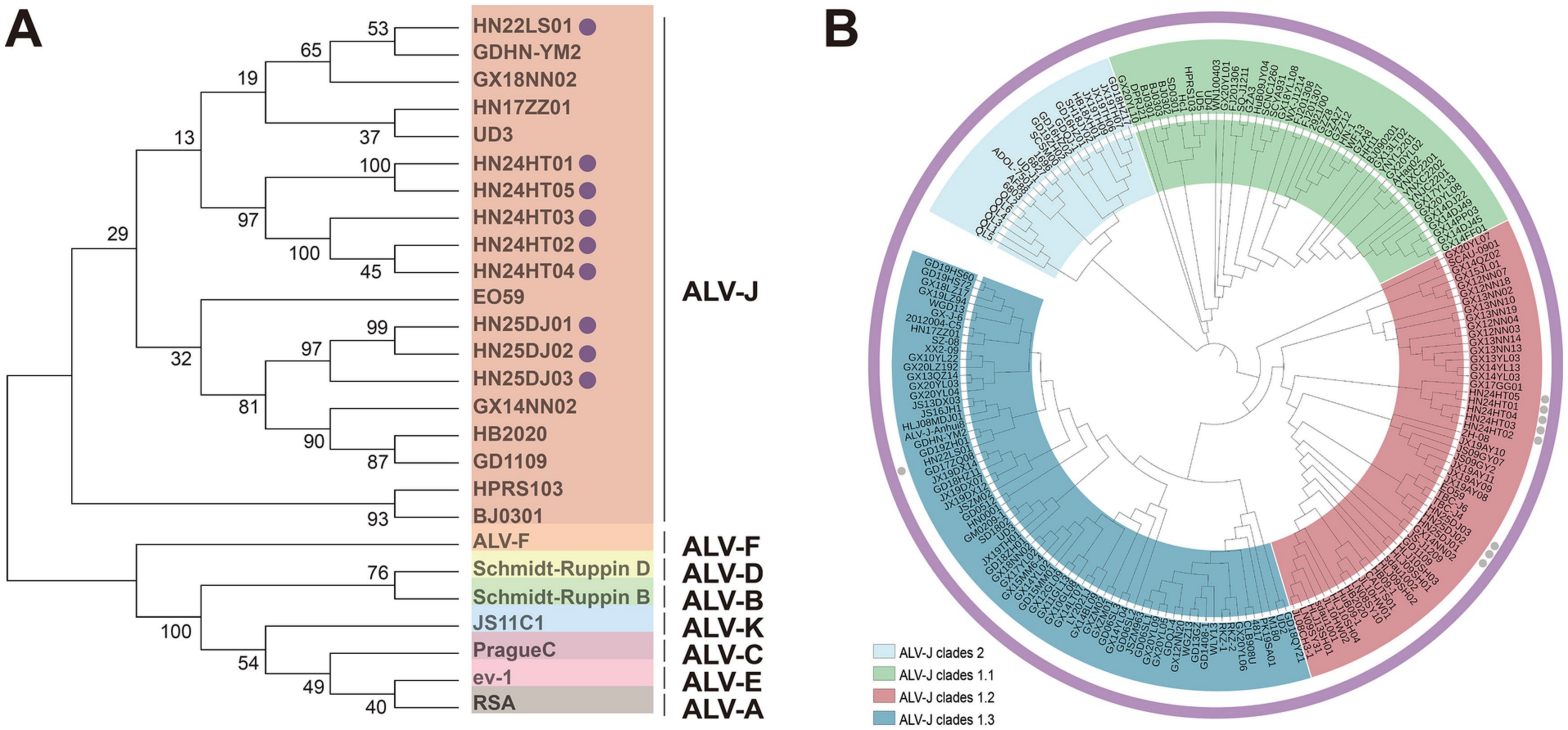
Phylogenetic analysis of ALV-J isolates. **(A)** Phylogenetic analysis for the Env protein of ALV-J isolates. Solid cycle (●) indicates ALV isolates in this study. The Neighbor-Joining method with 1,000 bootstrap replicates was used to infer the evolutionary relationships (MEGA 12). **(B)** Phylogenetic analysis for the Gp85 protein of ALV-J isolates. Solid cycle (●) indicates ALV isolates in this study. The Neighbor-Joining method with 1,000 bootstrap replicates was used to infer the evolutionary relationships (MEGA 12).

Phylogenetic analysis of ALV-J *env* demonstrated that the *env* genes of these nine isolates clustered in the same branch as the ALV-J reference strains, while other subgroups were grouped into different evolutionary branches (Figure 3A). These data clearly showed that these nine isolates belonged to ALV-J. Further phylogenetic analysis of *gp85* indicated that 8 ALV-J isolates derived from cockfighting and Hetian chickens clustered into the ALV-J Clades 1.2 branch, and the 1 ALV-J isolate from Lushi chickens clustered into the ALV-J clades 1.3 branch (Figure 3B). The isolates HN25DJ01, HN25DJ02, and HN25DJ03 isolated from cockfighting had a very close genetic distance to the Jiangsu isolates TBC-J4 and TBC-J6 as well as the Nigeria isolate EO59. The isolates HN24HT01, HN24HT02, HN24HT03, HN24HT04, and HN24HT05 isolated from Hetian chickens had a very close genetic distance to the Guangxi isolate GX17GG01 isolated in 2017. The isolate HN22LS01 isolated from Lushi chickens had a very close genetic distance to the Guangdong isolates GD19ZH01 and GDHN - YM2.

### 3.3 Characteristics of the Gp85 subunit of the isolates

**Figure 4:**
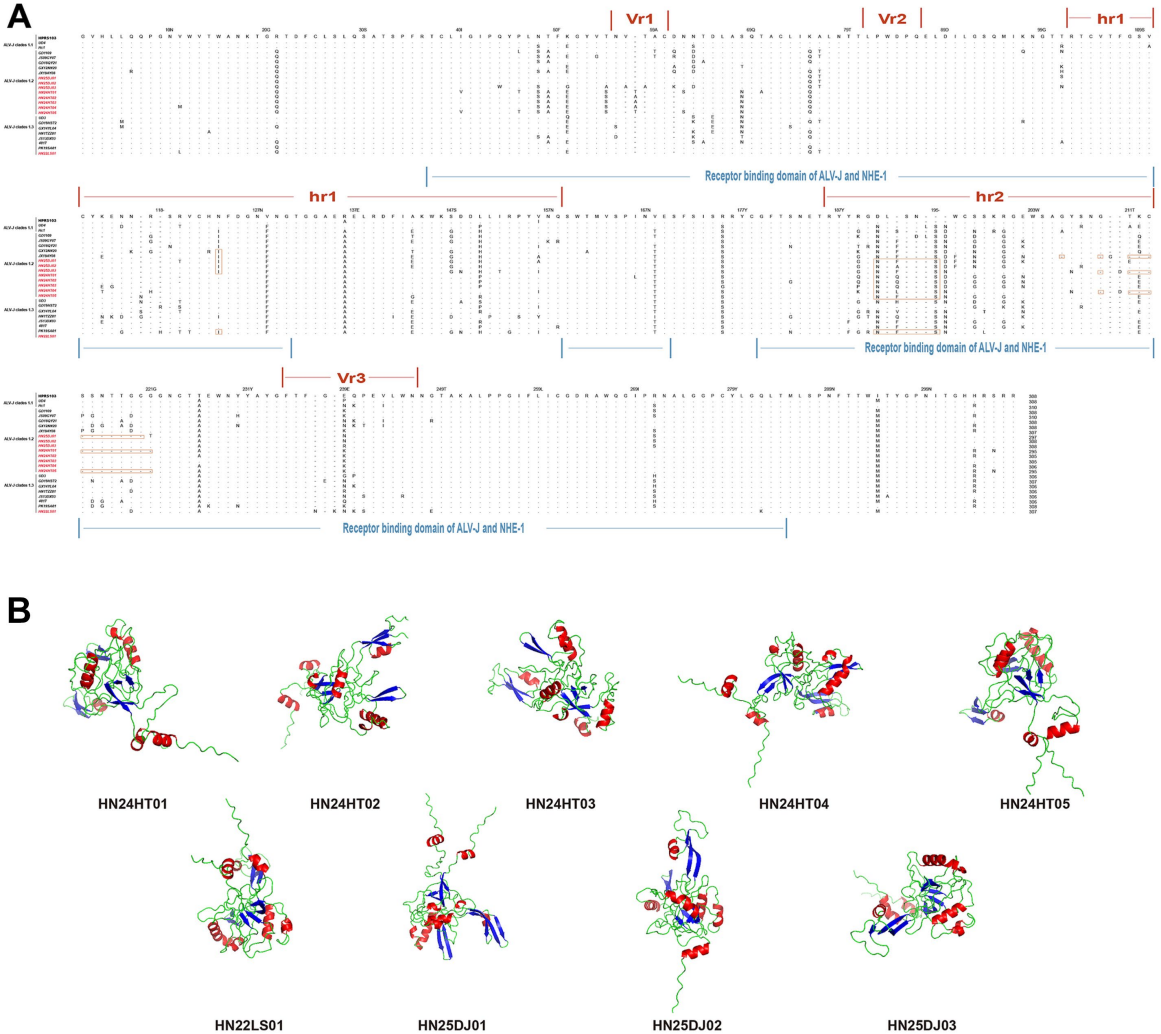
Amino acid sequence analysis and secondary structure prediction of ALV-J Gp85 protein. **(A)** Comparison of the amino acid sequences of Gp85 from the 9 ALV-J isolates and the ALV-J reference strains. The inferred Gp85 was compared to other ALV strains. The colored underlines represent the hr1, hr2, vr1, vr2, and vr3 regions and the receptor binding domain of ALV-J Gp85. The letters indicate amino acid substitutions, dots (.) indicate identical amino acid, and dashes (−) indicate gaps produced in the alignment. The 9 ALV-J isolates were marked in red. **(B)** Secondary structure of Gp85 protein analyzed using AlphaFold3. Red represents the alpha helix, and blue represents the beta sheet.

When comparing the Gp85 amino acid sequences of the isolated strains with those of the ALV-J reference strain, it was found that the mutations in all isolated strains mainly occurred in vr1, hr1, hr2, vr2, and vr3 (Figure 4A). Remarkable deletion mutations within the receptor-binding domain (RBD) were observed in HN25DJ01, HN24HT01, and HN24HT05, and these mutations partially overlapped with hr2. Specifically, compared with the ALV-J prototype strain HPRS-103, the deletion mutations of HN25DJ01 occurred at amino acid positions 206 and from 210 to 220, while those of HN24HT01 and HN24HT05 were located from position 210 to 221. Moreover, the N123I mutation in HN25DJ01, HN25DJ02, HN25DJ03, HN24HT01, and HN22LS01 enhanced the viral replication by increased Gp85 stability and an expanded interaction interface. Notably, the D191N mutation observed in all isolated strains compared with HPRS-103 suggested the emergence of a novel N-glycosylation site in the Env protein. Furthermore, using structural predictions generated by AlphaFold 3, we conducted a comparative analysis of the Gp85 proteins of nine ALV-J isolated strains to analyze the differences in their secondary structures. As depicted in Figure 4B, all isolated strains consistently contained nine β-sheet structures, whereas the number of α-helices varied: HN25DJ01 possessed 9 α-helix structures, HN25DJ03 and HN22LS01 had 8 α-helix structures, and HN25DJ02, HN24HT01, HN24HT02, HN24HT03, HN24HT04, and HN24HT05 each contained 7 α-helix structures.

### 3.4 Characteristics of the 3′ UTR of the isolates

**Figure 5:**
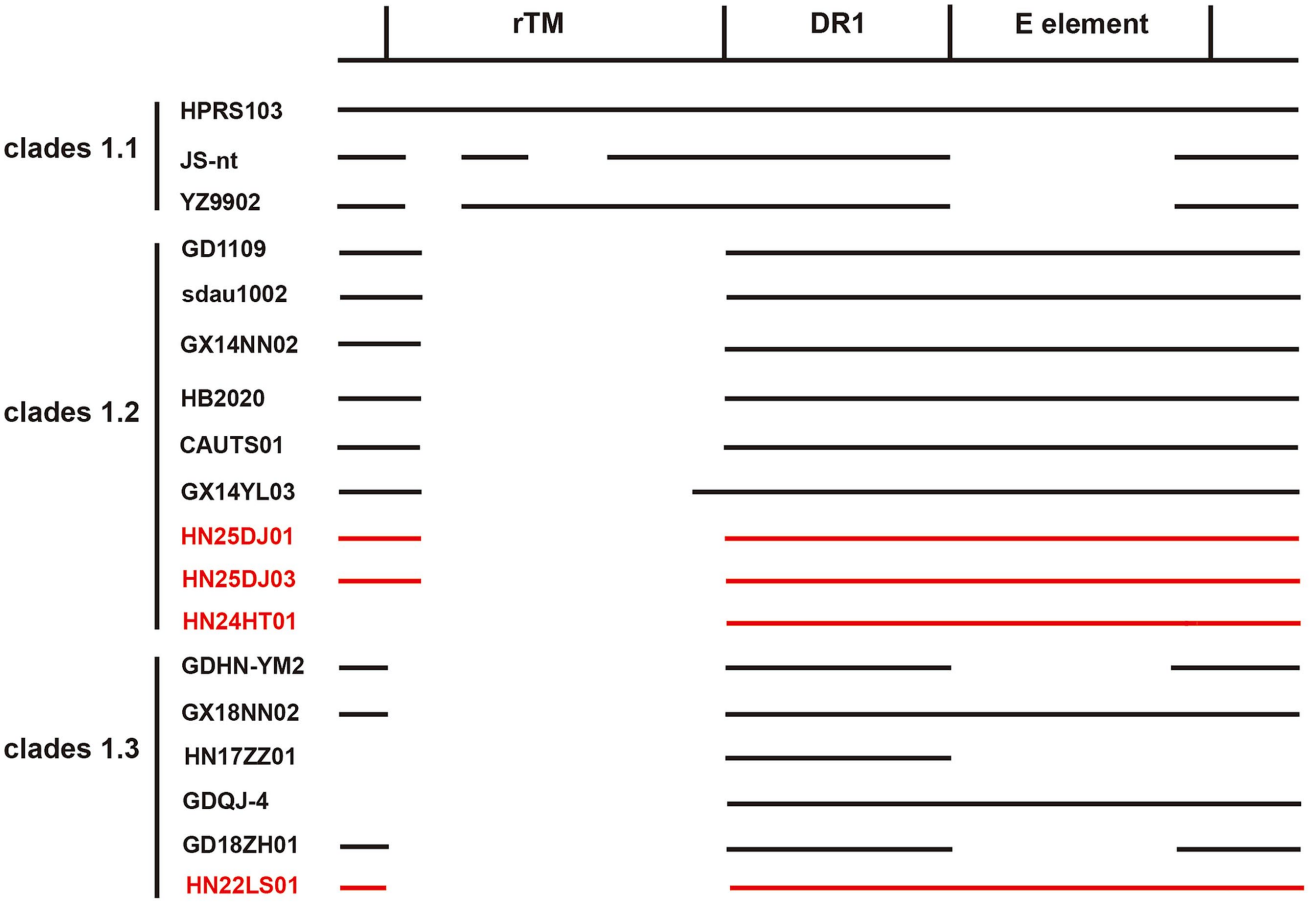
Comparison of the important regulatory elements of the 3′UTR regions of the isolates. The 4 ALV-J isolates were marked in red.

Genetic variations in the rTM, DR-1 and E elements within the 3′ UTR of ALV-J can influence the pathogenicity, oncogenicity and replication capacity. As shown in the Figure 5, the genetic variations within the 3′ UTR of the four isolated strains were relatively consistent. All of them retained the intact DR-1 and E elements and showed highest similarity to the reference strains GD1109, HB2020, CAUTS01 and sadu1002. The four isolated strains exhibited varying degrees of deletions in the r-TM region. HN25DJ01 and HN25DJ03 each had a deletion of 176 base pairs in the r-TM sequence, while HN24HT01 and HN22LS01 had a deletion of 210 base pairs in the r-TM sequence.

### 3.5 Transcription factor analysis of 3′LTR

**Figure 6:**
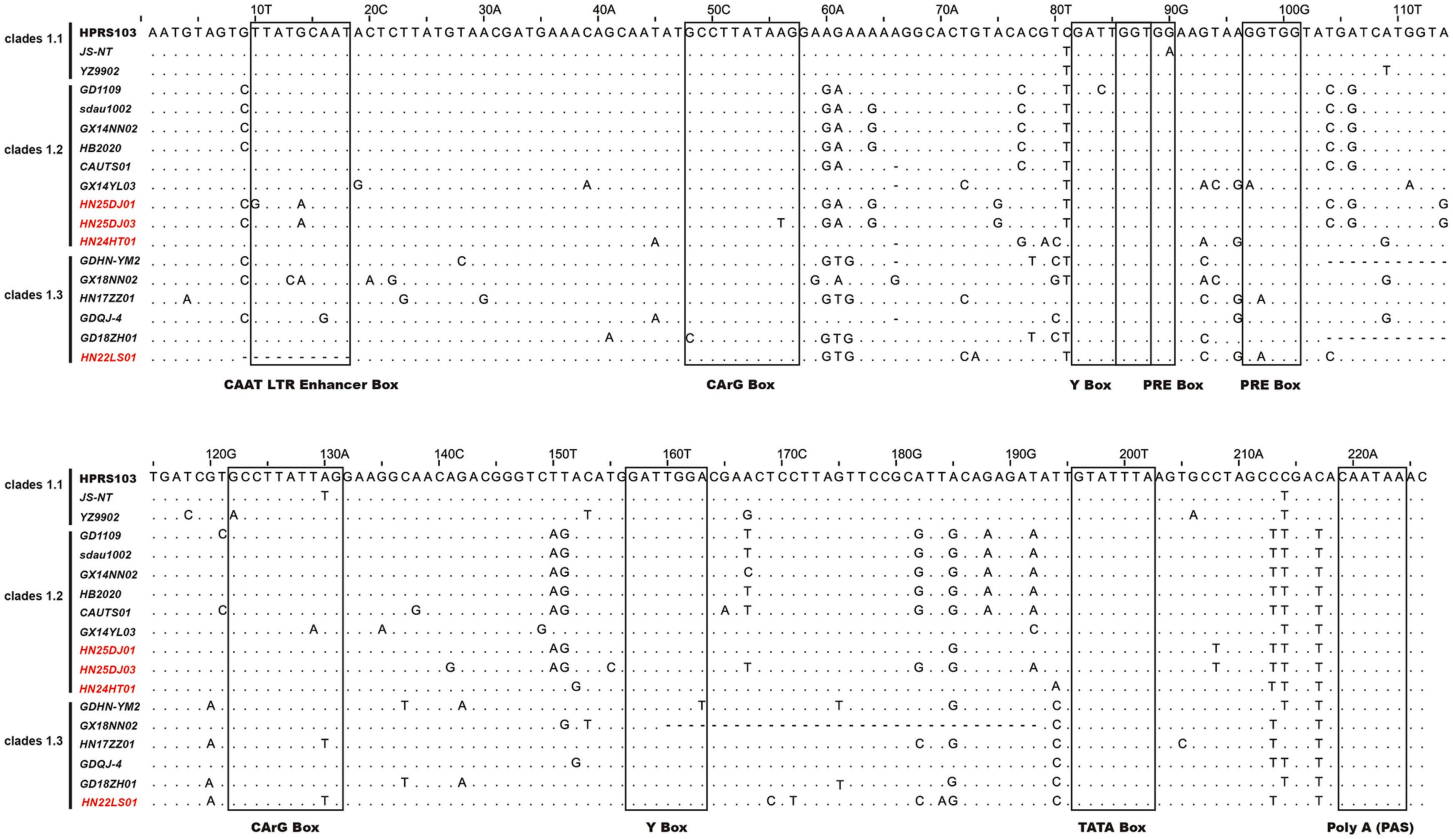
Analysis of the nucleotide sequences in the U3 region of ALV-J isolates. Letters indicate nucleotide substitutions; dots represent identical nucleotides; dashed lines indicate gaps produced in the alignment. The putative transcriptional regulatory elements are represented by black boxes.

Analysis of the LTR U3 region sequences and their transcription factor elements revealed that, compared with the 226 bp U3 region of the HPRS103 strain, the HN24HT01 isolate exhibited a 1 bp deletion at position 66, while the HN22LS01 isolate harbored a 10 bp deletion spanning positions 9 ~ 18 (Figure 6). Except for HN22LS01, which lacked one CAAT enhancer, all isolates contained two CArG boxes, Y boxes, PRE motifs, as well as a single CAAT enhancer, C/EBP box, and TATA box.

## 4 Discussion

Among the known ALV subgroups, ALV-J represents the greatest ongoing threat to China’s poultry industry due to its heightened pathogenicity and transmissibility (Liang et al., 2019; Tan et al., 2024). Since 2008, outbreaks of this virus have occurred in China’s layer chicken flocks and spread rapidly among various chicken breeds in China. To date, it has not been completely eradicated (Gao et al., 2012; Pan et al., 2012; Lin et al., 2016; Yang et al., 2025). This situation is largely because of its continuous evolution, which generates novel mutations affecting viral infection, replication, and pathogenicity (Cui et al., 2003; Zhang et al., 2025). Therefore, ongoing surveillance of the prevalent strains ALV-J and analysis of their molecular biological characteristics are of vital importance for the prevention and control of ALV in China and for the sustainable development of the poultry industry.

In this study, we successfully isolated and identified 9 strains of ALV-J (HN22LS01, HN24HT01, HN24HT02, HN24HT03, HN24HT04, HN24HT05, HN24DJ01, HN24DJ02, HN24DJ03) from native chickens in China. Compared with the ALV-J strains that have been published in GenBank, the homology of the *gp85* gene of the 9 strains of ALV-J was between 85.5 and 96.8%. Phylogenetic analysis of the Gp85 protein showed that all the isolated strains belonged to ALV-J Clade 1.2 and Clade 1.3. Consistent with the research results in recent years, compared with the initial ALV-J Clade 1.1 strains that entered China, Clade 1.2 and Clade 1.3 demonstrated a higher prevalence (Deng et al., 2021; Deng et al., 2022). The ALV-J isolates from cockfighting exhibited high homology with the isolates TBC-J4 and TBC-J6 from Jiangsu, as well as EO59 from Nigeria in Africa. The ALV-J isolates from Hetian chickens showed high homology with the isolate GX17GG01 from Guangxi. The ALV-J isolate from Lushi chickens were highly homologous to the isolates GDHN-YM2 and GD19ZH01 from Guangdong. The high homology between the Chinese isolates and the African isolates implies that ALV-J might have achieved cross-regional transmission through channels like transnational live chicken trade (Deng et al., 2022). This will help trace the global diffusion trajectory of ALV-J. Based on the homology, global monitoring can focus on the shared mutation features and collaboratively adjust prevention and control strategies.

Through in-depth analysis of the sequence and structural characteristics of the Gp85 protein, we found that significant differences existed in the functional domains and secondary structures among different isolates. Previous studies have confirmed that the two discontinuous amino acid sequences, namely positions 38–131 and 159–283 of the ALV-J Gp85 protein, constitute the RBD of Gp85 (Zhang et al., 2020). Continuous deletion mutations within the RBD of HN25DJ01 occurred at the amino acid positions from 210 to 220, while the deletion mutations of HN24HT01 and HN24HT05 were located at the amino acid positions from 210 to 221, potentially changing the efficiency of the binding between the virus and the host cell receptors and further impacting their replications (Federspiel, 2019). In addition, the N123I mutation occurring in HN25DJ01, HN25DJ02, HN25DJ03, HN24HT01, and HN22LS01 can enhance the replication ability of the virus by strengthening the stability of Gp85 and expanding the interaction interface (Yu et al., 2024). Mutations occurring at the 191st amino acid position in all isolated strains might lead to the formation of a new glycosylation site “NWS,” which could potentially result in changes in the epitope pattern of the env protein as well as in the replication of the virus (Xu et al., 2022). Secondary structure prediction analysis reveals that the number of α-helices varies among different isolates, which might have an impact on the interaction between the virus and its host (Guarracino et al., 2019). It also indicates that the Gp85 protein located on the surface of the ALV-J virion may enhance its adaptability to the host through conformational remodeling (Wang et al., 2025).

Through its three important elements (DR-1, E element, and r-TM), the 3′ UTR of ALV-J can influence the tissue preference of the virus and act as an enhancer to promote viral replication (Xu et al., 2023). Intriguingly, the ALV-J strain containing △-r-TM-type 3′ UTR exhibited stronger replication capacity and pathogenicity (Xu et al., 2023; Wang et al., 2012). Meanwhile, the integrity of the E element further enhances the replicative capacity of the virus (Cao et al., 2024). This type of 3′ UTR endows the virus with the strongest competitive advantage during the evolutionary process (Xu et al., 2023). In this study, the analysis of the 3′ UTR revealed that 4 ALV-J isolates (HN22LS01, HN24HT01, HN24DJ01, HN24DJ03) were similar to the majority of ALV-J isolates in that they all had deletions at r-TM while retaining the integrity of the E element. Therefore, compared with ALV-J with other types of 3′ UTR, we hypothesize that these four ALV-J isolates may possess enhanced replication and pathogenicity, which awaits further verification.

The U3 region contains promoters and enhancers, which are closely related to viral replication, transcription and translation, and are also involved in the activation of host proto-oncogenes (Zachow and Conklin, 1992; Cullen et al., 1985). In this study, a unique 10 bp deletion was observed in the U3 region of the HN22LS01 ALV-J isolate. This mutation led to the loss of the CAAT enhancer box transcription factor, which was not detected in the other three isolates. The CAAT enhancer box can enhance the replication ability of ALV-J (Gao et al., 2015). Therefore, compared with other 3 isolates, it is hypothesized that HN22LS01 may have a weaker replication ability.

In conclusion, our study isolated and characterized nine ALV-J strains from three different breeds of native chickens in China during the period from 2022 to 2025, and analyzed the molecular biological characteristics. The observed structural and genetic changes, particularly in *gp85* and the 3′UTR, indicate that these viruses may have evolved enhanced replication capacity and pathogenicity, which are closely related to the genetic evolution of ALV-J. In terms of disease prevention and control, ALV-J with enhanced replication capacity increases the difficulty of prevention and control. Its stronger pathogenicity causes more severe poultry diseases and enables rapid virus spread, so monitoring needs to be strengthened using more sensitive detection methods, and stricter biosecurity measures must be adopted (Cheng et al., 2023). In terms of vaccination, the possible contamination caused by the ALV-J with stronger replication capacity and pathogenicity during the production of commercial live vaccines may lead to immunization failure and greater losses (Wang et al., 2021). In terms of breeding programs, developing individuals with genetic resistance to ALV-J requires complex gene editing. Meanwhile, comprehensive analyses must be conducted to assess the potential impact of gene editing on ALV-J evolution (Matoušková et al., 2024). In the future, continuous monitoring of ALV-J variation, investigation of its epidemiology, and efforts to control and eradicate ALV-J strains in China will be essential.

## Data Availability

The datasets presented in this study can be found in online repositories. The names of the repository/repositories and accession number(s) can be found in the article/Supplementary material.

## Footnotes

^1^ https://deepmind.google/technologies/alphafold/alphafold-server/

## Supplementary material

Supplementary Figure S1 The PCR result of primers specific targeting ALV. M: Maker, 1: DNA of liver samples from Lushi chickens, 2: Isolate serum from anticoagulated blood to infect DF-1 cells, 3: DNA of liver samples from the first batch of cockfighting, 4: DNA of liver samples from the second batch of cockfighting, 5: Negative control, 6: Positive control. **(A)** ALV-A **(B)** ALV-B **(C)** ALV-K.

## References

[ref9001] Abramson J. Adler J. Dunger J. (2024). Accurate structure prediction of biomolecular interactions with AlphaFold3. Nature. 630, 493–500. doi: 10.1038/s41586-024-07487-w, PMID: 38718835 PMC11168924

[ref1] Böhnlein S. Hauber J. Cullen B. R. (1989). Identification of a U5-specific sequence required for efficient polyadenylation within the human immunodeficiency virus long terminal repeat. J. Virol. 63, 421–424. doi: 10.1128/JVI.63.1.421-424.1989, PMID: 2908926 PMC247699

[ref3] Cao Y. Ren Q. Chang S. Cui W. Zhao P. Wang Y. (2024). N6-methyladenosine RNA methylation modification regulates the transcription of viral-derived E (XSR) miRNAs to promote ALV-J replication. Vet. Microbiol. 298:110218. doi: 10.1016/j.vetmic.2024.110218 39159504

[ref4] Chen J. Li J. Dong X. Liao M. Cao W. (2022). The key amino acid sites 199-205, 269, 319, 321 and 324 of ALV-K env contribute to the weaker replication capacity of ALV-K than ALV-A. Retrovirology 19:19. doi: 10.1186/s12977-022-00598-0, PMID: 36002842 PMC9400301

[ref5] Chen X. Wang H. Fang X. Gao K. Fang C. Gu Y.. (2020). Identification of a novel epitope specific for Gp85 protein of avian leukosis virus subgroup K. Vet. Immunol. Immunopathol. 230:110143. doi: 10.1016/j.vetimm.2020.110143, PMID: 33129191

[ref6] Cheng X. Yang J. Bi X. Yang Q. Zhou D. Zhang S.. (2023). Molecular characteristics and pathogenicity of a Tibet-origin mutant avian leukosis virus subgroup j isolated from Tibetan chickens in China. Infect. Genet. Evol. 109:105415. doi: 10.1016/j.meegid.2023.105415 36775048

[ref7] Chesters P. M. Smith L. P. Nair V. (2006). E (XSR) element contributes to the oncogenicity of avian leukosis virus (subgroup J). J. Gen. Virol. 87, 2685–2692. doi: 10.1099/vir.0.81884-0, PMID: 16894209

[ref8] Cui Z. Du Y. Zhang Z. Silva R. F. (2003). Comparison of Chinese field strains of avian leukosis subgroup J viruses with prototype strain HPRS-103 and United States strains. Avian Dis. 47, 1321–1330. doi: 10.1637/6085, PMID: 14708978

[ref9] Cullen B. R. Raymond K. Ju G. (1985). Functional analysis of the transcription control region located within the avian retroviral long terminal repeat. Mol. Cell. Biol. 5, 438–447. doi: 10.1128/mcb.5.3.438-447.1985, PMID: 2985953 PMC366735

[ref10] Deng Q. Li M. He C. Lu Q. Gao Y. Li Q.. (2021). Genetic diversity of avian leukosis virus subgroup J (ALV-J): toward a unified phylogenetic classification and nomenclature system. Virus Evol. 7:veab037. doi: 10.1093/ve/veab037, PMID: 34026272 PMC8129623

[ref11] Deng Q. Li Q. Li M. Zhang S. Wang P. Fu F.. (2022). The emergence, diversification, and transmission of subgroup J avian leukosis virus reveals that the live chicken trade plays a critical role in the adaption and endemicity of viruses to the yellow-chickens. J. Virol. 96:e0071722. doi: 10.1128/jvi.00717-22, PMID: 35950858 PMC9472763

[ref12] Du Y. Ai Q. (1999). Detection of subgroup J avian leukosis virus in market broilers. Chin. J. Poultry Sci. 3, 1–4. (in Chinese)

[ref13] Fadly A. M. Smith E. J. (1999). Isolation and some characteristics of a subgroup J-like avian leukosis virus associated with myeloid leukosis in meat-type chickens in the United States. Avian Dis. 43, 391–400. doi: 10.2307/1592636, PMID: 10494407

[ref14] Fandiño S. Gomez-Lucia E. Benítez L. Doménech A. (2023). Avian leukosis: will we be able to get rid of it? Animals (Basel) 13:2358. doi: 10.3390/ani13142358, PMID: 37508135 PMC10376345

[ref15] Federspiel M. J. (2019). Reverse engineering provides insights on the evolution of subgroups a to E avian sarcoma and leukosis virus receptor specificity. Viruses 11:497. doi: 10.3390/v11060497, PMID: 31151254 PMC6630264

[ref16] Feng W. Zhou D. Meng W. Li G. Zhuang P. Pan Z.. (2017). Growth retardation induced by avian leukosis virus subgroup J associated with down-regulated Wnt/β-catenin pathway. Microb. Pathog. 104, 48–55. doi: 10.1016/j.micpath.2017.01.013, PMID: 28065818

[ref17] Freick M. Schreiter R. Weber J. Vahlenkamp T. W. Heenemann K. (2022). Avian leukosis virus (ALV) is highly prevalent in fancy-chicken flocks in Saxony. Arch. Virol. 167, 1169–1174. doi: 10.1007/s00705-022-05404-y, PMID: 35301570 PMC8964621

[ref18] Fung Y. K. Lewis W. G. Crittenden L. B. Kung H. J. (1983). Activation of the cellular oncogene c-erbB by LTR insertion: molecular basis for induction of erythroblastosis by avian leukosis virus. Cell 33, 357–368. doi: 10.1016/0092-8674(83)90417-8, PMID: 6305505

[ref19] Gao Y. Guan X. Liu Y. Li X. Yun B. Qi X.. (2015). An avian leukosis virus subgroup J isolate with a Rous sarcoma virus-like 5′-LTR shows enhanced replication capability. J. Gen. Virol. 96, 150–158. doi: 10.1099/vir.0.071290-0, PMID: 25274857

[ref20] Gao Y. Yun B. Qin L. Pan W. Qu Y. Liu Z.. (2012). Molecular epidemiology of avian leukosis virus subgroup J in layer flocks in China. J. Clin. Microbiol. 50, 953–960. doi: 10.1128/JCM.06179-11, PMID: 22205787 PMC3295186

[ref21] Guarracino D. A. Riordan J. A. Barreto G. M. Oldfield A. L. Kouba C. M. Agrinsoni D. (2019). Macrocyclic control in Helix mimetics. Chem. Rev. 119, 9915–9949. doi: 10.1021/acs.chemrev.8b00623, PMID: 31045350

[ref22] Li Y. Cui S. Li W. Wang Y. Cui Z. Zhao P.. (2017). Vertical transmission of avian leukosis virus subgroup J (ALV-J) from hens infected through artificial insemination with ALV-J infected semen. BMC Vet. Res. 13:204. doi: 10.1186/s12917-017-1122-4, PMID: 28662658 PMC5492345

[ref23] Li T. Yao X. Li C. Zhang J. Xie Q. Wang W.. (2020). Gp37 regulates the pathogenesis of avian leukosis virus subgroup J via its C terminus. J. Virol. 94, e02180–e02119. doi: 10.1128/JVI.02180-19 32213616 PMC7269434

[ref24] Liang X. Gu Y. Chen X. Li T. Gao Y. Wang X.. (2019). Identification and characterization of a novel natural recombinant avian leucosis virus from Chinese indigenous chicken flock. Virus Genes 55, 726–733. doi: 10.1007/s11262-019-01695-7, PMID: 31396785

[ref25] Liao L. Chen W. Zhang X. Zhang H. Li A. Yan Y.. (2022). Semen extracellular vesicles mediate vertical transmission of subgroup J avian leukosis virus. Virol. Sin. 37, 284–294. doi: 10.1016/j.virs.2022.01.026, PMID: 35527223 PMC9170978

[ref26] Lin W. Li X. Dai Z. Zhang X. Chang S. Zhao P.. (2016). Molecular epidemiology of J-subgroup avian leukosis virus isolated from meat-type chickens in southern China between 2013 and 2014. Arch. Virol. 161, 3039–3046. doi: 10.1007/s00705-016-3003-8, PMID: 27503348

[ref27] Liu P. Li L. Jiang Z. Yu Y. Chen X. Xiang Y.. (2021). Molecular characteristics of subgroup J avian leukosis virus isolated from yellow breeder chickens in Guangdong, China, during 2016-2019. Infect. Genet. Evol. 89:104721. doi: 10.1016/j.meegid.2021.104721, PMID: 33444858

[ref28] Malhotra S. Justice J. 4th Lee N. Li Y. Zavala G. Ruano M.. (2015). Complete genome sequence of an American avian leukosis virus subgroup j isolate that causes hemangiomas and myeloid leukosis. Genome Announc. 3:e01586-14. doi: 10.1128/genomeA.01586-14 25858851 PMC4392163

[ref29] Matoušková M. Plachý J. Kučerová D. Pecnová Ľ. Reinišová M. Geryk J.. (2024). Rapid adaptive evolution of avian leukosis virus subgroup J in response to biotechnologically induced host resistance. PLoS Pathog. 20:e1012468. doi: 10.1371/journal.ppat.1012468, PMID: 39146367 PMC11349186

[ref30] Meng F. Li X. Fang J. Gao Y. Zhu L. Xing G.. (2016). Genomic diversity of the avian leukosis virus subgroup J gp85 gene in different organs of an infected chicken. J. Vet. Sci. 17, 497–503. doi: 10.4142/jvs.2016.17.4.497, PMID: 27456778 PMC5204027

[ref31] Meng F. Li Q. Han R. Xu G. Gao X. Luo F.. (2022). A study on the infection status and transmission of avian leukosis virus subgroup J in Hy-line brown roosters. Arch. Virol. 167, 1521–1527. doi: 10.1007/s00705-022-05452-4, PMID: 35606465

[ref32] Mothes W. Boerger A. L. Narayan S. Cunningham J. M. Young J. A. (2000). Retroviral entry mediated by receptor priming and low pH triggering of an envelope glycoprotein. Cell 103, 679–689. doi: 10.1016/s0092-8674(00)00170-7, PMID: 11106737

[ref33] Ogert R. A. Lee L. H. Beemon K. L. (1996). Avian retroviral RNA element promotes unspliced RNA accumulation in the cytoplasm. J. Virol. 70, 3834–3843. doi: 10.1128/JVI.70.6.3834-3843.1996, PMID: 8648719 PMC190260

[ref34] Pan W. Gao Y. Qin L. Ni W. Liu Z. Yun B.. (2012). Genetic diversity and phylogenetic analysis of glycoprotein GP85 of ALV-J isolates from mainland China between 1999 and 2010: coexistence of two extremely different subgroups in layers. Vet. Microbiol. 156, 205–212. doi: 10.1016/j.vetmic.2011.10.019, PMID: 22101092

[ref35] Payne L. N. Brown S. R. Bumstead N. Howes K. Frazier J. A. Thouless M. E. (1991). A novel subgroup of exogenous avian leukosis virus in chickens. J. Gen. Virol. 72, 801–807. doi: 10.1099/0022-1317-72-4-801 1849967

[ref36] Payne L. N. Gillespie A. M. Howes K. (1993). Recovery of acutely transforming viruses from myeloid leukosis induced by the HPRS-103 strain of avian leukosis virus. Avian Dis. 37, 438–450. doi: 10.2307/1591671, PMID: 8395801

[ref37] Payne L. N. Nair V. (2012). The long view: 40 years of avian leukosis research. Avian Pathol. 41, 11–19. doi: 10.1080/03079457.2011.646237, PMID: 22845317

[ref38] Ruddell A. (1995). Transcription regulatory elements of the avian retroviral long terminal repeat. Virology 206, 1–7. doi: 10.1016/s0042-6822(95)80013-1, PMID: 7831764

[ref39] Su Q. Li Y. Cui Z. Chang S. Zhao P. (2018). The emerging novel avian leukosis virus with mutations in the pol gene shows competitive replication advantages both *in vivo* and *in vitro*. Emerg. Microbes Infect. 7:117. doi: 10.1038/s41426-018-0111-4, PMID: 29946141 PMC6018675

[ref40] Sun S. Cui Z. (2007). Epidemiological and pathological studies of subgroup J avian leukosis virus infections in Chinese local "yellow" chickens. Avian Pathol. 36, 221–226. doi: 10.1080/03079450701332345, PMID: 17497335

[ref41] Tan L. Li J. Duan Y. Liu J. Zheng S. Liang X.. (2024). Current knowledge on the epidemiology and prevention of avian leukosis virus in China. Poult. Sci. 103:104009. doi: 10.1016/j.psj.2024.104009, PMID: 39002365 PMC11298916

[ref42] Wang Q. Gao Y. Wang Y. Qin L. Qi X. Qu Y.. (2012). A 205-nucleotide deletion in the 3′ untranslated region of avian leukosis virus subgroup J, currently emergent in China, contributes to its pathogenicity. J. Virol. 86, 12849–12860. doi: 10.1128/JVI.01113-12, PMID: 22993155 PMC3497689

[ref43] Wang P. Li M. Li H. Bi Y. Lin L. Shi M.. (2021). ALV-J-contaminated commercial live vaccines induced pathogenicity in three-yellow chickens: one of the transmission routes of ALV-J to commercial chickens. Poult. Sci. 100:101027. doi: 10.1016/j.psj.2021.101027, PMID: 33647716 PMC7921873

[ref44] Wang Z. Wang S. Li J. Miao T. Luo Y. Shen T.. (2025). Isolation and molecular characteristic of subgroup J avian leukosis virus in Guangxi and Jiangsu provinces of China during 2022-2023. Poult. Sci. 104:105272. doi: 10.1016/j.psj.2025.105272, PMID: 40367569 PMC12141838

[ref45] Xu B. Dong W. Yu C. He Z. Lv Y. Sun Y.. (2004). Occurrence of avian leukosis virus subgroup J in commercial layer flocks in China. Avian Pathol. 33, 13–17. doi: 10.1080/03079450310001636237a, PMID: 14681063

[ref46] Xu M. Qian K. Shao H. Yao Y. Nair V. Ye J.. (2022). Glycosylation of ALV-J envelope protein at sites 17 and 193 is pivotal in the virus infection. J. Virol. 96:e0154921. doi: 10.1128/JVI.01549-21, PMID: 34878920 PMC8865534

[ref47] Xu M. Qian K. Shao H. Yao Y. Nair V. Ye J.. (2023). 3' UTR of ALV-J can affect viral replication through promoting transcription and mRNA nuclear export. J. Virol. 97:e0115223. doi: 10.1128/jvi.01152-23, PMID: 37902396 PMC10688361

[ref48] Yang N. Chen C. Xu M. Zhang G. (2025). Isolation and identification of avian leukosis virus subgroup J from Luohé Ma chicken. Virus Genes. doi: 10.1007/s11262-025-02174-y, PMID: 40627086

[ref49] Yu M. Zhang Y. Zhang L. Wang S. Liu Y. Xu Z.. (2024). N123I mutation in the ALV-J receptor-binding domain region enhances viral replication ability by increasing the binding affinity with chNHE1. PLoS Pathog. 20:e1011928. doi: 10.1371/journal.ppat.1011928, PMID: 38324558 PMC10878525

[ref50] Zachow K. R. Conklin K. F. (1992). CArG, CCAAT, and CCAAT-like protein binding sites in avian retrovirus long terminal repeat enhancers. J. Virol. 66, 1959–1970. doi: 10.1128/JVI.66.4.1959-1970.1992, PMID: 1312613 PMC288984

[ref51] Zhang F. Li H. Lin C. Wei Y. Zhang W. Wu Y.. (2024). Detection and genetic diversity of subgroup K avian leukosis virus in local chicken breeds in Jiangxi from 2021 to 2023. Front. Microbiol. 15:1341201. doi: 10.3389/fmicb.2024.1341201, PMID: 38389530 PMC10882074

[ref52] Zhang R. Mu W. Dong L. Luo S. Zhang S. Yao R.. (2025). Molecular characteristics of avian leukosis viruses isolated from indigenous chicken breeds in Yunnan Province, southwestern China. Poult. Sci. 104:104850. doi: 10.1016/j.psj.2025.104850, PMID: 39874784 PMC11810833

[ref53] Zhang Y. Yu M. Xing L. Liu P. Chen Y. Chang F.. (2020). The bipartite sequence motif in the N and C termini of gp85 of subgroup J avian leukosis virus plays a crucial role in receptor binding and viral entry. J. Virol. 94, e01232–e01220. doi: 10.1128/JVI.01232-20 32878894 PMC7592230

[ref54] Zheng L. P. Teng M. Li G. X. Zhang W. K. Wang W. D. Liu J. L.. (2022). Current epidemiology and co-infections of avian immunosuppressive and neoplastic diseases in chicken flocks in Central China. Viruses 14:2599. doi: 10.3390/v14122599, PMID: 36560601 PMC9784009

