# Supplementary figures and images for: Isolation and molecular characterization of subgroup J avian leukosis virus in native chicken breeds of China during 2022–2025
Source: Front Microbiol. 2025 Oct 6;16:1684812. doi: 10.3389/fmicb.2025.1684812 (PMC12536225; doi:10.3389/fmicb.2025.1684812)

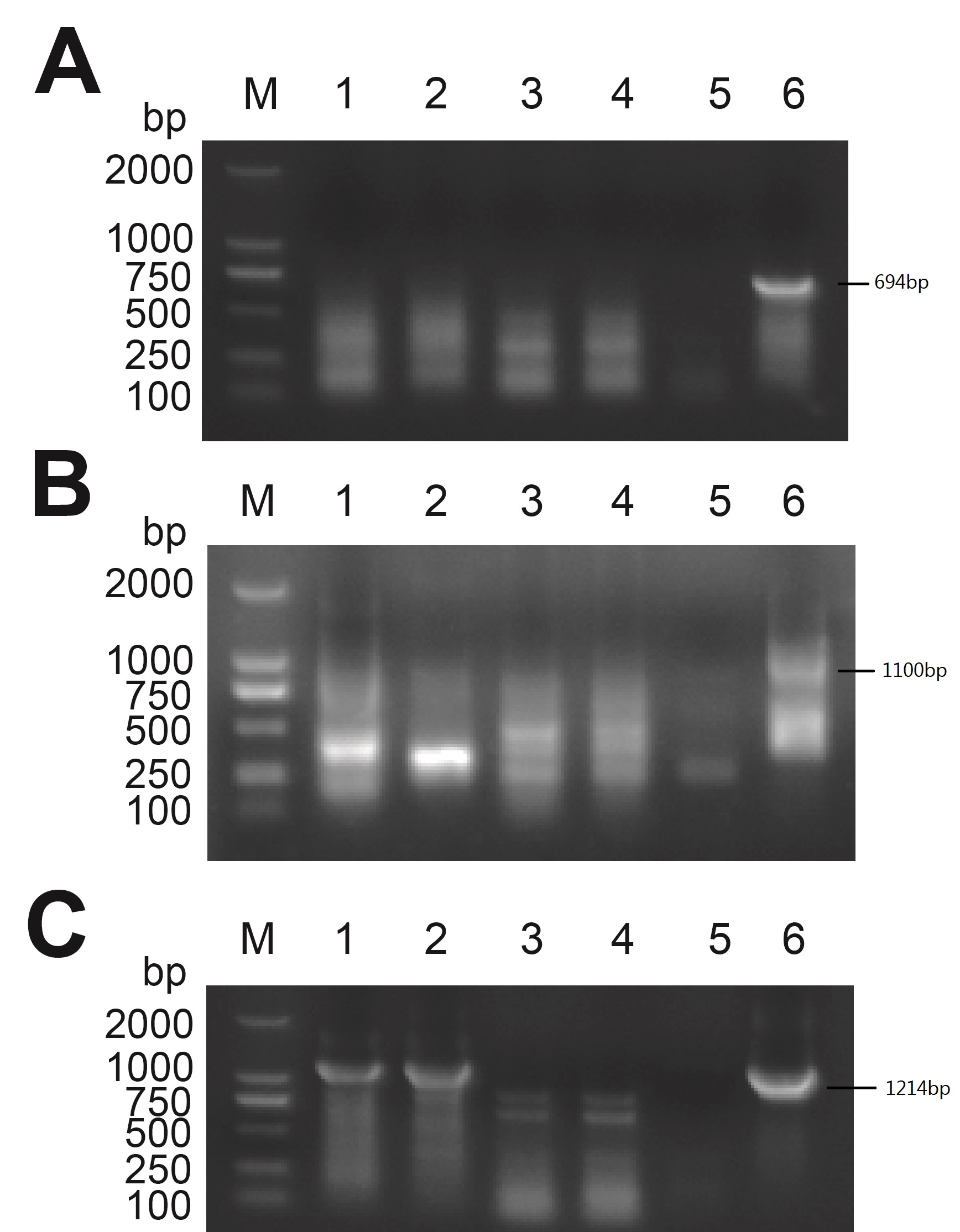

Supplement: Supplementary Figure S1 — The PCR result of primers specific targeting ALV. M: Maker, 1: DNA of liver samples from Lushi chickens, 2: Isolate serum from anticoagulated blood to infect DF-1 cells, 3: DNA of liver samples from the first batch of cockfighting, 4: DNA of liver samples from the second batch of cockfighting, 5: Negative control, 6: Positive control. (A) ALV-A (B) ALV-B (C) ALV-K. [file Image_1.JPEG]
