# Supplementary material for: Isolation and molecular characterization of subgroup J avian leukosis virus in native chicken breeds of China during 2022–2025
Source: Front Microbiol. 2025 Oct 6;16:1684812. doi: 10.3389/fmicb.2025.1684812 (PMC12536225; doi:10.3389/fmicb.2025.1684812)
Supplement: Supplementary file 2 [file Table_1.DOCX]

**Supplementary Table1. ALV strains used in this study**

| **No.** | **Strains** | **Origin** | **Year** | **Subgroup** | **Accession no.** |
| --- | --- | --- | --- | --- | --- |
| 1 | RSA | FRA | 1990 | ALV-A | M37980 |
| 2 | Schmidt-Ruppin B | USA | 1998 | ALV-B | AF052428 |
| 3 | Prague C | USA | 1982 | ALV-C | J02342 |
| 4 | Schmidt-Ruppin D | Japan | 1992 | ALV-D | D10652 |
| 5 | ev-1 | USA | 2000 | ALV-E | AY013303 |
| 6 | ALV-F | USA | 2004 | ALV-F | AY608692 |
| 7 | JS11C1 | Shandong,CHN | 2011 | ALV-K | KF746200 |
| 8 | AF88 | USA | 1997 | ALV-J | AF247390 |
| 9 | ADOL-7501 | USA | 1997 | ALV-J | AY027920 |
| 10 | UD-J1 | USA | 2000 | ALV-J | AF305091 |
| 11 | 6827 | USA | 1997 | ALV-J | AF247389 |
| 12 | 1696 | USA | 1997 | ALV-J | AF247384 |
| 13 | SCSM00 | Sichuan,CHN | 2011 | ALV-J | KF796652 |
| 14 | GD19ZH02 | Guangdong,CHN | 2019 | ALV-J | MT538249 |
| 15 | GD16HZ02 | Guangdong,CHN | 2016 | ALV-J | MT538252 |
| 16 | GDQJ-1 | Guangdong,CHN | 2014 | ALV-J | KU254611 |
| 17 | GD16HZ01 | Guangdong,CHN | 2016 | ALV-J | MT538250 |
| 18 | SH18JY02 | Shanghai,CHN | 2018 | ALV-J | MN735307 |
| 19 | HB18XH01 | Hebei,CHN | 2018 | ALV-J | MN735298 |
| 20 | JX19TH09 | Jiangxi,CHN | 2019 | ALV-J | MN262536 |
| 21 | JX19TH06 | Jiangxi,CHN | 2019 | ALV-J | MN262533 |
| 22 | JX19TH07 | Jiangxi,CHN | 2019 | ALV-J | MN262534 |
| 23 | GD18HZ17 | Guangdong,CHN | 2018 | ALV-J | MN262615 |
| 24 | BJ0301 | Beijing,CHN | 2003 | ALV-J | AY897230 |
| 25 | BJ0303 | Beijing,CHN | 2003 | ALV-J | AY897232 |
| 26 | BJ0302 | Beijing,CHN | 2003 | ALV-J | AY897231 |
| 27 | SD0301 | Shandong,CHN | 2003 | ALV-J | AY897228 |
| 28 | Hc1 | USA | 1993 | ALV-J | AF097731 |
| 29 | HPRS103 | UK | 1988 | ALV-J | Z46390 |
| 30 | UD5 | USA | 2000 | ALV-J | AF307952 |
| 31 | UD4 | USA | 2000 | ALV-J | AF307951 |
| 32 | WN100403 | Shandong,CHN | 2010 | ALV-J | HQ333257 |
| 33 | FJ201306 | Fujian,CHN | 2013 | ALV-J | KM655820 |
| 34 | SQ-J1211 | Jiangsu,CHN | 2012 | ALV-J | KC282895 |
| 35 | GZA3 | Shandong,CHN | 2016 | ALV-J | KX010989 |
| 36 | HuB09JY04 | Heilongjiang,CHN | 2009 | ALV-J | JN378888 |
| 37 | SCNC1260 | Sichuan,CHN | 2011 | ALV-J | KF796650 |
| 38 | SCYA931 | Sichuan,CHN | 2011 | ALV-J | KF796656 |
| 39 | GX18YL108J | Guangxi,CHN | 2018 | ALV-J | MZ393152 |
| 40 | WX-J1214 | Jiangsu,CHN | 2012 | ALV-J | KC417026 |
| 41 | FJ201308 | Fujian,CHN | 2013 | ALV-J | KM655822 |
| 42 | FJ201307 | Fujian,CHN | 2013 | ALV-J | KM655821 |
| 43 | 2921/00 | Taibei,CHN | 2000 | ALV-J | EF653908 |
| 44 | GZA27 | Shandong,CHN | 2016 | ALV-J | KX010990 |
| 45 | GZZ8 | Shandong,CHN | 2016 | ALV-J | KX010991 |
| 46 | GZZ12 | Shandong,CHN | 2016 | ALV-J | KX010992 |
| 47 | WF13 | Guangdong,CHN | 2013 | ALV-J | KJ631314 |
| 48 | GZA8 | Shandong,CHN | 2016 | ALV-J | KX077606 |
| 49 | HH11 | Heilongjiang,CHN | 2011 | ALV-J | JX014438 |
| 50 | BJ090201 | Beijing,CHN | 2009 | ALV-J | HQ333067 |
| 51 | AHaq02 | Anhui,CHN | 2012 | ALV-J | KF534753 |
| 52 | GX13LT02 | Guangxi,CHN | 2013 | ALV-J | KY983559 |
| 53 | GX17YL33 | Guangxi,CHN | 2017 | ALV-J | MZ393151 |
| 54 | GX14DJ49 | Guangxi,CHN | 2014 | ALV-J | KY983566 |
| 55 | GX14PP03 | Guangxi,CHN | 2014 | ALV-J | KX506774 |
| 56 | GX14FF01 | Guangxi,CHN | 2014 | ALV-J | KU848765 |
| 57 | GX14DJ45 | Guangxi,CHN | 2014 | ALV-J | KY983565 |
| 58 | GX14DJ22 | Guangxi,CHN | 2014 | ALV-J | KY983564 |
| 59 | GX14QZ02 | Guangxi,CHN | 2014 | ALV-J | KT598472 |
| 60 | GX17GG01 | Guangxi,CHN | 2017 | ALV-J | MW491258 |
| 61 | GX15JL01 | Guangxi,CHN | 2015 | ALV-J | MN735294 |
| 62 | GX12NN07 | Guangxi,CHN | 2012 | ALV-J | KT598487 |
| 63 | GX12NN18 | Guangxi,CHN | 2012 | ALV-J | KT598485 |
| 64 | GX13NN02 | Guangxi,CHN | 2013 | ALV-J | KT598482 |
| 65 | GX13NN10 | Guangxi,CHN | 2013 | ALV-J | KT598481 |
| 66 | GX12NN04 | Guangxi,CHN | 2012 | ALV-J | KT598488 |
| 67 | GX12NN03 | Guangxi,CHN | 2012 | ALV-J | KT598489 |
| 68 | GX13NN14 | Guangxi,CHN | 2013 | ALV-J | KT598479 |
| 69 | GX13NN13 | Guangxi,CHN | 2013 | ALV-J | KT598480 |
| 70 | GX13YL03 | Guangxi,CHN | 2013 | ALV-J | KT598476 |
| 71 | GX14YL13 | Guangxi,CHN | 2014 | ALV-J | KT598468 |
| 72 | GX14YL03 | Guangxi,CHN | 2014 | ALV-J | KR025484 |
| 73 | GX18QY21 | Guangxi,CHN | 2018 | ALV-J | MN262619 |
| 74 | ZH-08 | Guangdong,CHN | 2008 | ALV-J | HQ316166 |
| 75 | JX19AY10 | Jiangxi,CHN | 2019 | ALV-J | MN262549 |
| 76 | JS09GY07 | Jiangsu,CHN | 2009 | ALV-J | HM595734 |
| 77 | JS09GY2 | Jiangsu,CHN | 2009 | ALV-J | GU982307 |
| 78 | JX19AY11 | Jiangxi,CHN | 2019 | ALV-J | MN262550 |
| 79 | JX19AY09 | Jiangxi,CHN | 2019 | ALV-J | MN262548 |
| 80 | JX19AY08 | Jiangxi,CHN | 2019 | ALV-J | MN262547 |
| 81 | GX14NN02 | Guangxi,CHN | 2014 | ALV-J | MN735293 |
| 80 | JS-J1209 | Jiangsu,CHN | 2012 | ALV-J | KC282893 |
| 83 | HLJ10SH03 | Heilongjiang,CHN | 2010 | ALV-J | HQ634813 |
| 84 | HLJ09SH01 | Heilongjiang,CHN | 2009 | ALV-J | HQ634806 |
| 85 | GD1109 | Guangdong,CHN | 2011 | ALV-J | JX254901 |
| 86 | sadu1002 | Shandong,CHN | 2010 | ALV-J | JN389518 |
| 87 | HLJ09SH02 | Heilongjiang,CHN | 2009 | ALV-J | HQ634807 |
| 88 | HB09-1 | Beijing,CHN | 2009 | ALV-J | HM600812 |
| 89 | CAUTS01 | Beijing,CHN | 2009 | ALV-J | JF932002 |
| 90 | JL10HW01 | Heilongjiang,CHN | 2010 | ALV-J | HQ634800 |
| 91 | LN08SY10 | Heilongjiang,CHN | 2008 | ALV-J | HQ634802 |
| 92 | HB2020 | Hubei,CHN | 2020 | ALV-J | ON840093 |
| 93 | HuB09-1 | Hubei,CHN | 2009 | ALV-J | HM600813 |
| 94 | HLJ10SH04 | Heilongjiang,CHN | 2010 | ALV-J | HQ634814 |
| 95 | JL10HW02 | Heilongjiang,CHN | 2010 | ALV-J | HQ634801 |
| 96 | sadu1001 | Shandong,CHN | 2010 | ALV-J | JN389517 |
| 97 | HLJ13SH01 | Heilongjiang,CHN | 2013 | ALV-J | KM376510 |
| 98 | LN09SY31 | Heilongjiang,CHN | 2009 | ALV-J | HQ634803 |
| 99 | JL08CH3-1 | Heilongjiang,CHN | 2008 | ALV-J | HQ634809 |
| 100 | GD18ZH01 | Guangdong,CHN | 2018 | ALV-J | MT538243 |
| 101 | HN0001 | Henan,CHN | 2001 | ALV-J | AY897219 |
| 102 | GD0512 | Guangdong,CHN | 2005 | ALV-J | EF103133 |
| 103 | GD19ZH01 | Guangdong,CHN | 2019 | ALV-J | MT538248 |
| 104 | GDHN-YM2 | Guangdong,CHN | 2018 | ALV-J | MK683479 |
| 105 | EO59 | Nigeria | 2017 | ALV-J | MF926336 |
| 106 | HN17ZZ01 | Henan,CHN | 2017 | ALV-J | MN735299 |
| 107 | ALV-J-Anhui8 | Anhui,CHN | 2018 | ALV-J | MH186094 |
| 108 | HLJ08MDJ01 | Heilongjiang,CHN | 2008 | ALV-J | HQ634810 |
| 109 | JS16JH1 | Jiangsu,CHN | 2016 | ALV-J | MG700533 |
| 110 | JS13DX03 | Jiangsu,CHN | 2013 | ALV-J | KM873177 |
| 111 | GD06SL2 | Guangdong,CHN | 2006 | ALV-J | EF103129 |
| 112 | GX14YL04 | Guangxi,CHN | 2014 | ALV-J | KT598469 |
| 113 | 2012004-C5 | Guangxi,CHN | 2012 | ALV-J | KC453974 |
| 114 | GD06SL3 | Guangdong,CHN | 2006 | ALV-J | EF103130 |
| 115 | GD18HZ11 | Guangdong,CHN | 2018 | ALV-J | MN262609 |
| 116 | GX-J-6 | Guangxi,CHN | 2010 | ALV-J | JQ246095 |
| 117 | WGD13 | Guangdong,CHN | 2013 | ALV-J | KJ631312 |
| 118 | GX19LZ94 | Guangxi,CHN | 2019 | ALV-J | MW491260 |
| 119 | GX18LZ17 | Guangxi,CHN | 2018 | ALV-J | MW491259 |
| 120 | GD19HS72J | Guangdong,CHN | 2019 | ALV-J | MZ393164 |
| 121 | GD19HS60J | Guangdong,CHN | 2019 | ALV-J | MZ393165 |
| 122 | SCAU-0901 | Guangdong,CHN | 2008 | ALV-J | FJ619190 |
| 123 | SZ-08 | Guangdong,CHN | 2008 | ALV-J | HM775329 |
| 124 | PK19SA01 | Pakistan | 2018 | ALV-J | MN956380 |
| 125 | 4817 | USA | 1996 | ALV-J | AF247385 |
| 126 | CLB908U | Russia | 2009 | ALV-J | JQ935966 |
| 127 | UD3 | USA | 2000 | ALV-J | AF307950 |
| 128 | SD1802 | Shandong,CHN | 2018 | ALV-J | MN419336 |
| 129 | GM0209-1 | Shandong,CHN | 2018 | ALV-J | MH379642 |
| 130 | M180 | Guangdong,CHN | 2016 | ALV-J | KX611834 |
| 131 | GX12NN02 | Guangxi,CHN | 2012 | ALV-J | KT598483 |
| 132 | GDQJ-4 | Guangdong,CHN | 2014 | ALV-J | KU170199 |
| 133 | GD06SL1 | Guangdong,CHN | 2006 | ALV-J | EF107624 |
| 134 | WLY13 | Guangdong,CHN | 2013 | ALV-J | KJ631311 |
| 135 | GD1408-1 | Guangdong,CHN | 2014 | ALV-J | KU500036 |
| 136 | GD13GZ | Guangdong,CHN | 2013 | ALV-J | KU500030 |
| 137 | WGZ13 | Guangdong,CHN | 2013 | ALV-J | KJ631313 |
| 138 | GX10YL22 | Guangxi,CHN | 2010 | ALV-J | KT598497 |
| 139 | XX2-09 | Guangdong,CHN | 2009 | ALV-J | HM775331 |
| 140 | 2921/00 | Guangdong,CHN | 2010 | ALV-J | HM775330 |
| 141 | GX20YL03 | Guangxi,CHN | 2020 | ALV-J | MW491264 |
| 142 | GX14BL09 | Guangxi,CHN | 2014 | ALV-J | KT598473 |
| 143 | GX14LT07 | Guangxi,CHN | 2014 | ALV-J | KX034517 |
| 144 | GX13QZ14 | Guangxi,CHN | 2013 | ALV-J | KT598477 |
| 145 | GX10GL08 | Guangxi,CHN | 2010 | ALV-J | KT598499 |
| 146 | GX11GL13 | Guangxi,CHN | 2011 | ALV-J | KT598493 |
| 147 | GX12GL09 | Guangxi,CHN | 2012 | ALV-J | KT598490 |
| 148 | GD15MM01 | Guangdong,CHN | 2015 | ALV-J | MN066152 |
| 149 | GX14YL02 | Guangxi,CHN | 2014 | ALV-J | KT598471 |
| 150 | GX18NN02 | Guangxi,CHN | 2018 | ALV-J | MN066140 |
| 151 | GX17YL02 | Guangxi,CHN | 2017 | ALV-J | MW491254 |
| 152 | GX15MM6-4 | Guangxi,CHN | 2015 | ALV-J | KU848763 |
| 153 | JS-nt | Jiangsu,CHN | 2016 | ALV-J | HM235667 |
| 154 | YZ9902 | Jiangsu,CHN | 2016 | ALV-J | HM235670 |
| 155 | QL5 | Egypt | 2019 | ALV-J | MN496125 |
| 156 | QL1 | Egypt | 2019 | ALV-J | MN496121 |
| 157 | QL3 | Egypt | 2018 | ALV-J | MN496123 |
| 158 | QL4 | Egypt | 2019 | ALV-J | MN496124 |
| 159 | QL6 | Egypt | 2018 | ALV-J | MN496126 |
| 160 | QL2 | Egypt | 2018 | ALV-J | MN496122 |
| 161 | 6803 | USA | 1997 | ALV-J | AF247388 |
| 162 | YNYL2201 | Yunnan,CHN | 2022 | ALV-J | PQ468438 |
| 163 | YNXC2201 | Yunnan,CHN | 2022 | ALV-J | PQ468439 |
| 164 | YNXC2202 | Yunnan,CHN | 2022 | ALV-J | PQ468441 |
| 165 | YNJC2201 | Yunnan,CHN | 2022 | ALV-J | PQ468440 |
| 166 | GXZM01 | Guangxi,CHN | 2025 | ALV-J | PV553666 |
| 167 | GXZM02 | Guangxi,CHN | 2025 | ALV-J | PV553667 |
| 168 | JSZM02 | Jiangsu,CHN | 2025 | ALV-J | PV553668 |
| 169 | JSZM963 | Jiangsu,CHN | 2025 | ALV-J | PV553669 |
| 170 | DPRJ21 | VRL | 2022 | ALV-J | [OK507207](https://www.ncbi.nlm.nih.gov/nuccore/OK507207) |
| 171 | LY2021J | Fujian,CHN | 2021 | ALV-J | OP918846 |
| 172 | RKZ-1 | Shandong,CHN | 2022 | ALV-J | OP508143 |
| 173 | HN-1 | Xinjiang,CHN | 2024 | ALV-J | PQ010741 |
| 174 | TBC-J4 | Jiangsu,CHN | 2020 | ALV-J | MT409624 |
| 175 | TBC-J6 | Jiangsu,CHN | 2020 | ALV-J | [MT409625](https://www.ncbi.nlm.nih.gov/nuccore/MT409625.1) |
| 176 | RKZ-2 | Shandong,CHN | 2022 | ALV-J | OP856678 |
| 177 | GX20YL10 | Guangxi,CHN | 2020 | ALV-J | MW491271 |
| 178 | GX20YL09 | Guangxi,CHN | 2020 | ALV-J | MW491270 |
| 179 | GX20YL08 | Guangxi,CHN | 2020 | ALV-J | MW491269 |
| 180 | GX20YL07 | Guangxi,CHN | 2020 | ALV-J | MW491268 |
| 181 | GX20YL06 | Guangxi,CHN | 2020 | ALV-J | MW491267 |
| 182 | GX20YL05 | Guangxi,CHN | 2020 | ALV-J | MW491266 |
| 183 | GX20YL04 | Guangxi,CHN | 2020 | ALV-J | MW491265 |
| 184 | GX20YL02 | Guangxi,CHN | 2020 | ALV-J | MW491263 |
| 185 | GX20YL01 | Guangxi,CHN | 2020 | ALV-J | MW491262 |
| 186 | **HN22LS01** | **Henan,CHN** | **2022** | **ALV-J** | **PX099218** |
| 187 | **HN24HT01** | **Fujian,CHN** | **2023** | **ALV-J** | **PX099219** |
| 188 | **HN24HT02** | **Fujian,CHN** | **2023** | **ALV-J** | **PX108887** |
| 189 | **HN24HT03** | **Fujian,CHN** | **2023** | **ALV-J** | **PX108888** |
| 190 | **HN24HT04** | **Fujian,CHN** | **2023** | **ALV-J** | **PX108889** |
| 191 | **HN24HT05** | **Fujian,CHN** | **2023** | **ALV-J** | **PX108890** |
| 192 | **HN24DJ01** | **Henan,CHN** | **2025** | **ALV-J** | **PX099220** |
| 193 | **HN24DJ02** | **Henan,CHN** | **2025** | **ALV-J** | **PX108891** |
| 197 | **HN24DJ03** | **Henan,CHN** | **2025** | **ALV-J** | **PX099221** |
